# Programmed cell death-ligand 1 expression predicts poor treatment response and prognostic value in esophageal squamous cell carcinoma patients without esophagectomy

**DOI:** 10.18632/aging.203326

**Published:** 2021-07-22

**Authors:** Fang Zhang, Xiuyuan Zhu, Qi Zhang, Ping Zhou, Liang Hao

**Affiliations:** 1 Department of Radiation Oncology, Yantai Affiliated Hospital of Binzhou Medical University, Yantai 26400, Shandong, China; 2 Department of Anesthesiology, The First Hospital of Zibo, Zibo 255200, Shandong, China; 3 Department of Pathology, The First Hospital of Zibo, Zibo 255200, Shandong, China

**Keywords:** esophageal cancer, squamous cell carcinoma, PD-L1, disease control rate, immunotherapy

## Abstract

Research on association between programmed cell death ligand 1 (PD-L1) expression in cancer cells and prognosis of esophageal squamous cell carcinoma (ESCC) has been controversial and has focused on patients with surgical resection. We aimed to investigate impact of PD-L1 on treatment response and prognostic value in ESCC and analyze which subset of patients may benefit from immunotherapy. The PD-L1 expression was evaluated by immunohistochemical analysis in all patients. Stratification analysis was performed according to whether surgery was performed. There were no significant correlations between PD-L1 expression with 3-year overall survival (OS) and progression-free survival (PFS) in 81 ESCC patients. Then stratification analysis was performed. Among these 44 patients without surgery, disease control rate (DCR) in negative PD-L1 expression group (78%) was significantly better than those (42%) in positive PD-L1 expression group (*P* = 0.032). There were no significant correlations between PD-L1 expression with 3-year OS and PFS in 37 ESCC patients receiving surgery. However, in 44 ESCC patients without surgery, the Kaplan-Meier method showed that 3-year OS and PFS in negative PD-L1 expression group were significantly better than those in positive PD-L1 expression group. In Cox univariate and multivariate model, PD-L1 was an independent prognosticator for inferior OS (*p* = 0.011; *p* = 0.017). Our research revealed prognostic role of PD-L1 expression in cancer cells may be variable in different treatment methods. Consequently, PD-L1 may serve as an independent prognostic factor and provide a theoretical basis for combining conventional therapy with immunotherapy targeting PD-L1 to achieve better treatment outcome in ESCC patients without esophagectomy.

## INTRODUCTION

According to 2018 global cancer statistics, the incidence of esophageal cancer (EC) ranks seventh, and the cancer-related mortality ranks sixth in the world [1]. Every year, almost half of the new cases diagnosed and deaths of EC in the world come from China, which is the country with the largest burden of EC [1]. In China, the mortality rate of EC is the fourth among cancer-related deaths, and esophageal squamous cell carcinoma (ESCC) accounts for more than 90% of cases [2, 3]. With the significant advancement of diagnostic and treatment technologies, the survival rates of EC had increased, but the 5-year Overall survival (OS) was still around 30% in China from 2003 to 2015 [4]. Currently, the immunotherapy had rapidly emerged as a novel treatment option, achieved very significant efficacy, and it was thought that it would turn into one of the main treatment methods of EC. Through the combination of programmed cell death-1 (PD-1) and programmed cell death-ligand 1 (PD-L1), it can promote tumor cells escape from host immune surveillance [5]. A series of clinical trials have confirmed that EC patients with immunotherapy targeting PD-1/PD-L1 can obtain good treatment response and long-lasting effects, but the number of effective patients is only 12% to 30%, indicating that screening the benefiting population is the key [6–9].

PD-L1 is called B7 homologous protein 1 and composed of five domains, which is a type I transmembrane protein. PD-L1 is constitutively expressed in antigen presenting cells, tumor cells, non-hematopoietic cells and non-lymphoid organs such as liver, heart and lung [10]. After PD-L1 in tumor cell binds to PD-1 receptor on T cell, it inhibits the migration and proliferation of T cell and helps tumor cell escape from host immune surveillance [11]. It alters the production and maturation of cytokines, and leads to T cells apoptosis, which contributes to the growth of cancer cells [10, 12].

Some studies have shown that the PD-L1 was a good prognostic factor of patients with ESCC [13, 14]. Conversely, some studies have observed that PD-L1 positive ESCC patients have a worse prognosis [15–17]. Additionally, some studies confirmed that there was no correlation between PD-L1 expression in cancer cells and survival for ESCC patients [18, 19]. All the above evidences about the association between PD-L1 in cancer cells and ESCC survival have concentrated on patients with esophagectomy and been controversial. However, prognostic value of PD-L1 in ESCC patients without surgery remains to be determined. We aimed to observe the impact of PD-L1 expression on treatment response and prognostic value in ESCC and analyze which part of patients are beneficiaries of immunotherapy. Further understanding of the influence of PD-L1 expression on prognosis is important for improving prognosis and screening the benefiting population from immunotherapy for ESCC patients.

## MATERIALS AND METHODS

### Patients

From January 2015 to December 2017, 81 patients who were primary ESCC were available for this study. Enrollment criteria include no history of other malignant tumors, pathologically confirmed ESCC, complete case data and follow-up information. The exclusion criteria were as follows: non-squamous cell carcinoma and second primary cancer. Consequently, 81 patients were recruited for the present study. Record clinical data, mainly including pathological data and basic characteristics of patients. According to the seventh edition of the AJCC TNM staging system, the selected patients were staged. We followed up all patients until death or 31 December 2020. OS was defined from the date of diagnosis to death due to any cause or the last follow-up. Progression-free survival (PFS) was defined as the time from start time of treatment to death from any cause or date of first relapse. Disease control rate (DCR) is defined as the percentage of patients whose tumors shrink or remain unchanged within a certain period of time, including complete remission, partial remission and stable cases. The patients who were alive until 31 December 2020 was defined as censored data. In our research, all procedures were carried out according to ethical principles, and the ethics committee of our hospital had approved the study.

### Immunohistochemistry

The patient’s tissue was fixed in 4% buffered formalin, embedded with paraffin, and finally cut into 3 μm-thick sections for immunohistochemistry (IHC) examination. Briefly, the section was dewaxed in xylene, rehydrated in descending grades of ethanol, soaked in distilled water for 10 min; and blocked for 30 min with 3% H2O2. After blocking, Antigen retrieval: 0.01M sodium citrate buffer solution (pH6.0), microwave intensity 100, microwave antigen retrieval 10min. After blocking at 37°C, incubate the sections with rabbit anti-PD-L1monoclonal antibody (1:60, AB205921, Abcam) at 4°C overnight. On the second day, add second antibody to the sections and incubate at room temperature for 60 min, then observe through DAB system staining and counterstain the sections with hematoxylin.

### Evaluation of immunostaining

Two experienced pathologists independently examined all specimens blindly. Analyze the slides semi-quantitatively, and give reports based on the relative staining percentage as previously described on tumor cells [20]. During the IHC analysis, we use positive controls in accordance with the instructions to ensure quality control. Five visual fields were selected for PD-L1 expression score. Calculate the average percentage of PD-L1 positive cells in the five fields of each sample. If PD-L1 staining of the tumor cell is >5%, it is considered that the expression of PD-L1 is positive.

### Statistical analysis

We use SPSS version 20.0 statistical package for data processing (SPSS Inc., Chicago, IL, USA). Fisher's exact test or Chi-square test was applied for categorical data between the two groups. We use Kaplan-Meier curve and log-rank analysis to analyze and compare the survival of patients. We applied the Cox proportional hazard model to conduct univariate and multivariate analysis to determine the hazard ratio (HR) of the variables to the survival. The 95% HR confidence interval (CI) was given. Two-sided test was performed. We considered that *P* < 0.05 was statistically significant.

### Ethical statement

The study was approved by the institutional ethics committee of Yantai Affiliated Hospital of Binzhou Medical University (Application number: 20200104001) and all procedures were conducted in accordance with ethical principles.

## RESULTS

### Patient's characteristics

**Figure 1:**
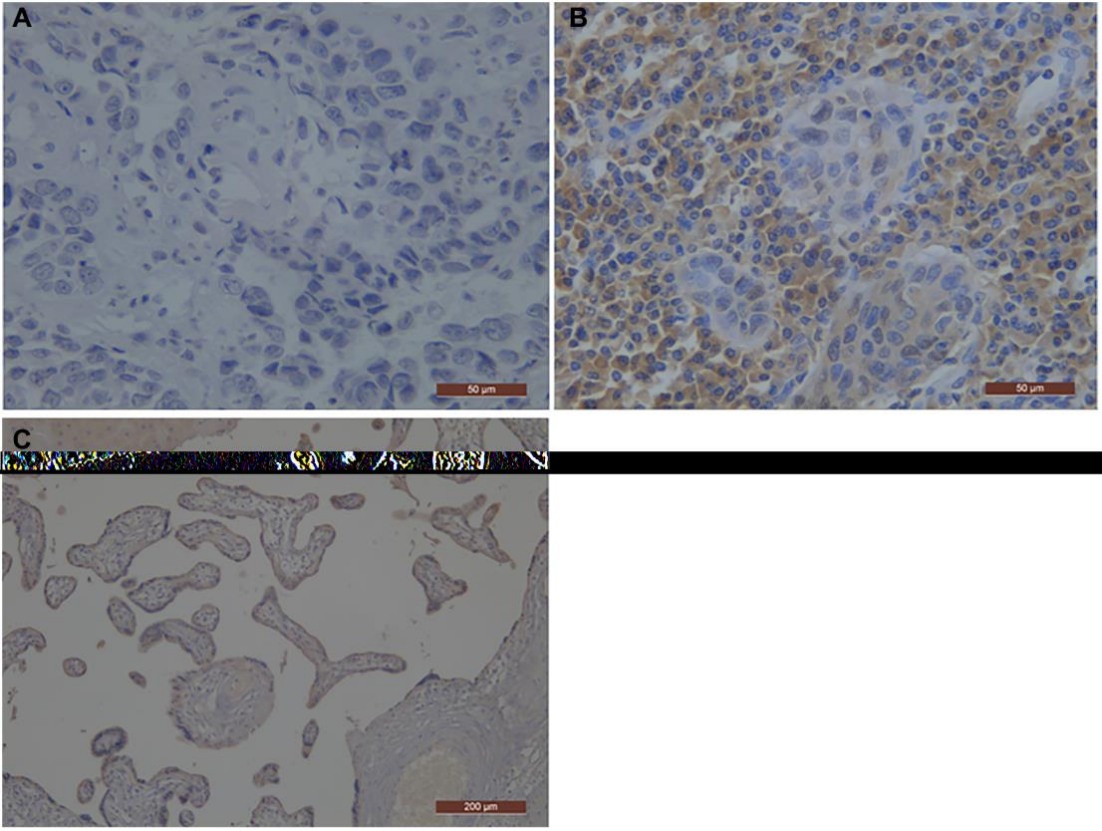
**Representative photographs of PD-L1 immunostaining in esophageal squamous cell carcinoma.** The positive staining was assessed against the positive control staining (Placental tissue). (**A**) Negative immunohistochemical staining pattern for PD-L1; (**B**) Positive immunohistochemical staining pattern for PD-L1; (**C**) the positive control staining (Placental tissue); PD-L1, programmed death-ligand 1.

In this study, we retrospectively analyzed 81 ESCC patients, including 75 males and 6 females. Based on the time the patient was diagnosed, the age range of the patients was 43 to 92 years, with a median age of 74 years. There were 37 patients receiving surgery and 44 patients without surgery. PD-L1 expression was positive in 36 (44%) of 81 patients, 24 (65%) of 37 patients, and 12 (27%) of 44 patients, respectively (Figure 1). 32 (40%) patients were I and II, and 49 (60%) patients were III and IV. Additionally, 34 (42%) patients had negative status of lymph nodes, and 47 (58%) patients had positive status of lymph nodes. The patient's primary tumor location was 23 cases (28%) in the upper esophagus, 32 cases (40%) in the middle esophagus, and 26 cases (32%) in the lower esophagus. Among the 81 patients, 28 (35%) patients were non-smokers, whereas 53 (65%) patients were smokers. Among the 81 patients, 32 (40%) patients were non-drinkers, whereas 49 (60%) patients were drinkers. Patient characteristics are presented in Table 1.

**Table 1:** Clinicopathologic features of 81 patients with esophageal squamous cell carcinoma.

**Parameters**		**No. of cases (Percentage)**
Age (years) (median:74, range 43–92)	≤65 >65	44 (54%) 37 (46%)
Sex	Male Female	75 (93%) 6 (7%)
TNM stage	I II III IV	32 (40%) 49 (60%)
Status of lymph nodes	Negative Positive	34 (42%) 47 (58%)
Primary tumor location	Upper Middle Lower	23 (28%) 32 (40%) 26 (32%)
PD-L1 expression	Negative Positive	45 (56%) 36 (44%)
Smoking history	No Yes	28 (35%) 53 (65%)
Alcohol history	No Yes	32 (40%) 49 (60%)
Surgery	No Yes	44 (54%) 37 (46%)

### The correlations between PD-L1 and patient characteristics.

Among all ESCC patients, there were no obvious relationships between PD-L1 and smoking history, alcohol history, age, sex, lymph node status, M stage and TNM stage. Although there were associations between PD-L1 and primary tumor location (*P* = 0.078) and T stage (*P* = 0.054), they did not achieve statistical significance (*P* ≥ 0.05) (Table 2).

**Table 2:** Correlations between PD-L1 expression and clinicopathologic parameters of 81 ESCC patients.

**Parameters**		**PD-L1**		
		**+**	**−**	***P* value**
Age (years)	≤65 >65	21 15	23 22	0.517
Sex	Male Female	32 4	43 2	0.399
Tumor location	Upper Middle Low	7 19 10	16 13 16	0.078
TNM stage	I II III IV	14 22	18 27	0.919
T stage	T1/2 T3/4	8 28	3 42	0.054
M stage	M0 M1	34 2	40 5	0.454
Status of lymph nodes	Negative Positive	13 23	21 24	0.339
Smoking history	Yes No	23 13	30 15	0.818
Alcohol history	Yes No	22 14	27 18	0.919

Groups were performed according to the operation, one group of 37 ESCC patients receiving surgery and one group of 44 ESCC patients without surgery. Similarly, there were no obvious associations between PD-L1 and smoking history, alcohol history, age, sex, tumor location, T stage, lymph node status, TNM stage and M stage in both groups (Tables 3 and 4).

**Table 3:** Correlations between PD-L1 expression and clinicopathologic parameters of 37 ESCC patients receiving surgery.

**Parameters**		**PD-L1**		
		**+**	**−**	***P* value**
Age (years)	≤65 >65	17 7	12 1	0.216
Sex	Male Female	22 2	13 0	0.532
Tumor location	Upper Middle Low	3 14 7	3 6 4	0.664
TNM stage	I II III IV	10 14	5 8	0.850
T stage	T1/2 T3/4	7 17	2 11	0.446
Status of lymph nodes	Negative Positive	7 17	4 9	0.919
Smoking history	Yes No	17 7	9 4	0.602
Alcohol history	Yes No	16 8	8 5	0.755

**Table 4:** Correlations between PD-L1 expression and clinicopathologic parameters of 44 ESCC patients without surgery.

**Parameters**		**PD-L1**		
		**+**	**−**	***P* value**
Age (years)	≤65 >65	4 8	11 21	0.621
Sex	Male Female	10 2	30 2	0.297
Tumor location	Upper Middle Low	4 5 3	13 7 12	0.412
TNM stage	I II III IV	4 8	13 19	0.739
T stage	T1/2/3 T4	7 5	26 6	0.139
M stage	M0 M1	11 1	28 4	0.583
Status of lymph nodes	Negative Positive	6 6	17 15	0.853
Smoking history	Yes No	6 6	21 11	0.489
Alcohol history	Yes No	6 6	19 13	0.576

### Associations between DCR with clinicopathological characteristics in 44 ESCC patients without surgery

From Table 5, we found that there were statistical associations between DCR with Tumor location (*P* = 0.046), TNM stage (*P* = 0.038) and PD-L1 expression (*P* = 0.032) in 44 ESCC patients without surgery. DCR (78%) in the PD-L1 negative group was significantly better than those (42%) in the PD-L1 positive group. However, there were no statistical correlations between DCR and smoking history, alcohol history, age, sex, T stage and status of lymph nodes.

**Table 5:** Associations between disease control rate with clinicopathological characteristics in 44 ESCC patients without surgery.

**Parameters**		**DCR**		
		**Present**	**Absent**	***P* value**
Age (years)	≤65 >65	8 22	7 7	0.759
Sex	Male Female	27 3	13 1	0.759
Tumor location	Upper Middle Low	15 6 9	2 7 5	0.046
TNM stage	I II III IV	14 16	2 12	0.038
T stage	T1/2/3 T4	24 6	9 5	0.287
Status of lymph nodes	Negative Positive	18 12	5 9	0.133
Smoking history	Yes No	16 14	11 3	0.184
Alcohol history	Yes No	15 15	10 4	0.211
PD-L1	+ −	5 25	7 7	0.032

### Stratification analysis between patient survival with clinicopathological characteristics

#### Survival analysis

**Figure 2:**
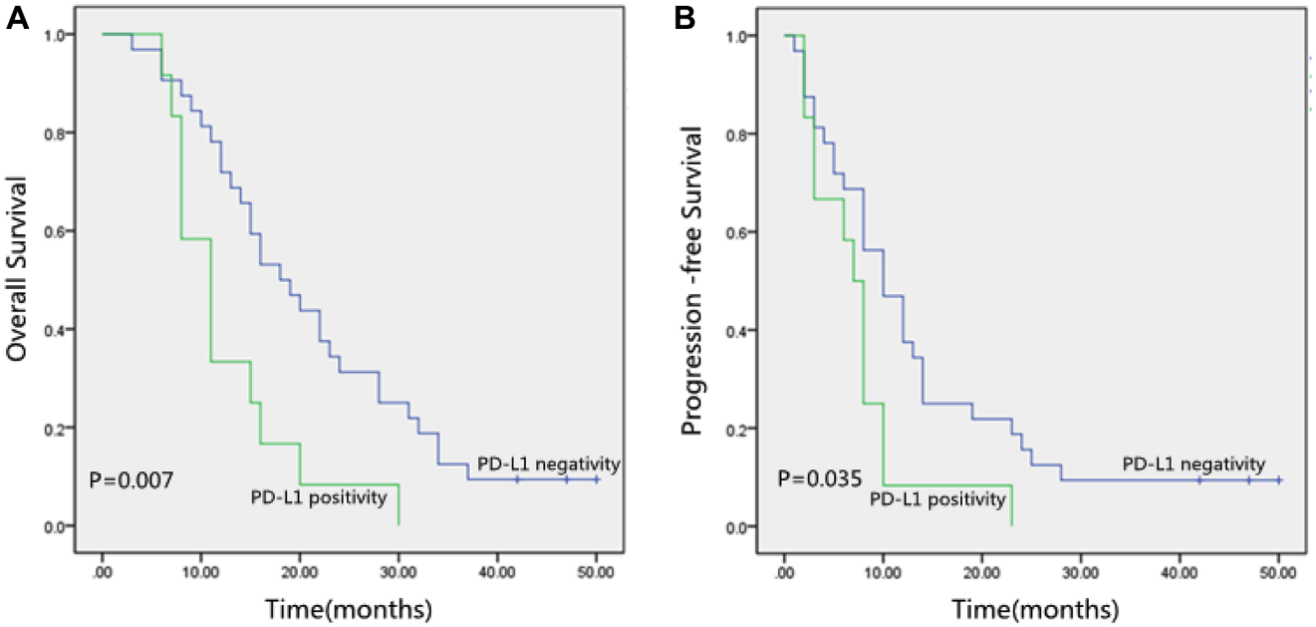
(**A**) Kaplan-Meier curves of Overall Survival (OS) according to PD-L1 expression. (**B**) Kaplan-Meier curves of Progression-Free Survival (PFS) according to PD-L1 expression. PD-L1, programmed death-ligand 1.

Table 6 revealed the relationship between 3-Year OS and PFS with PD-L1. There were no statistical associations between PD-L1 and 3-year OS and PFS in 81 ESCC patients (*P* > 0.05). Then stratification analysis was performed according to whether surgery was performed. There were no statistical associations between PD-L1 and 3-year OS and PFS in 37 ESCC patients receiving surgery (*P* > 0.05). However, the Kaplan-Meier method showed that 3-year OS and PFS in the PD-L1 negative group were obviously better than those in the PD-L1 positive group (*P* < 0.05; Table 6 and Figure 2) in 44 ESCC patients without surgery.

**Table 6:** Results of log-rank analysis between 3-Year OS and PFS with PD-L1 expression.

**Factors**		**No. of patients**	**3-Year OS *p* value**	**3-Year PFS *p* value**
PD-L1 of 81 ESCC patients	+ −	36 45	0.383	0.217
PD-L1 of 37 ESCC patients receiving surgery	+ −	24 13	0.583	0.298
PD-L1 of 44 ESCC patients without surgery	+ −	12 32	0.007	0.035

We observed that PD-L1 positive (*p* = 0.007; Figure 2A), TNM stage (*p* = 0.008), and Lymph nodes status (*p* = 0.001) were correlated with inferior OS significantly by the Kaplan-Meier method and log-rank test (Table 7). Besides, Table 7 also showed that PD-L1 positive (*p* = 0.035; Figure 2B), Tumor location (*p* = 0.049), TNM stage (*p* = 0.018), and Lymph nodes status (*p* = 0.044) were significantly correlated with poor PFS.

**Table 7:** Results of log-rank analysis of clinicopathologic parameters for 3-Year OS and PFS in 44 ESCC patients without surgery.

**Factors**		**No. of patients**	**3-Year OS *p* value**	**3-Year PFS *p* value**
Age (years)	≤65 >65	15 29	0.830	0.776
Sex	Male Female	40 4	0.991	0.533
Tumor location	Upper Middle Low	17 12 15	0.059	0.049
TNM stage	I II III IV	17 27	0.008	0.018
T stage	T1-3 T4	33 11	0.106	0.032
Status of lymph nodes	Negative Positive	23 21	0.001	0.044
Smoking history	Yes No	27 17	0.730	0.483
Alcohol history	Yes No	25 19	0.536	0.624
PD-L1	+ −	12 32	0.007	0.035

#### The cox univariate and multivariate analysis

For 44 ESCC patients without surgery, we performed the Cox Univariate and Multivariate Analysis (Tables 8 and 9). In the Cox univariate model, PD-L1 positive (*p* = 0.011, HR: 2.516, 95% CI: 1.234–5.127), TNM stage (*p* = 0.012, HR: 2.373, 95% CI: 1.207–4.663) and Status of lymph nodes (*p* = 0.002, HR: 2.944, 95% CI: 1.485–5.836) were independently poor prognosticators for inferior OS. Meanwhile, TNM stage (*p* = 0.029, HR: 2.085, 95% CI: 1.080–4.027) and T stage (*p* = 0.049, HR: 2.126, 95% CI: 1.004–4.504) were related with inferior PFS. Although they are not statistically significant, there was a trend between the positive PD-L1 expression with 3-Year PFS (*P* = 0.052). The positive PD-L1 was correlated with worse survival and increased risk of recurrence.

**Table 8:** Cox univariate analysis for 3-year survival in 44 patients ESCC patients without surgery.

	**3-Year OS**			**3-Year PFS**		
	**HR**	**95% CI**	***P***	**HR**	**95% CI**	***P***
Tumor location	1.246	0.881–1.761	0.213	1.108	0.787–1.560	0.557
TNM stage	2.373	1.207–4.663	0.012	2.085	1.080–4.027	0.029
T stage	1.745	0.863–3.527	0.121	2.126	1.004–4.504	0.049
Status of lymph nodes	2.944	1.485–5.836	0.002	1.815	0.973–3.385	0.061
PD-L1	2.516	1.234–5.127	0.011	2.008	0.993–4.058	0.052

**Table 9:** Cox multivariate analysis for 3-year survival in 44 patients ESCC patients without surgery.

	**3-Year OS**			**3-Year PFS**		
	**HR**	**95% CI**	***P***	**HR**	**95% CI**	***P***
Age	1.157	0.497–2.694	0.735	0.718	0.322–1.598	0.417
Sex	1.190	0.350–4.045	0.780	1.536	0.418–5.642	0.518
Tumor location	1.096	0.702–1.711	0.686	1.126	0.745–1.701	0.575
TNM stage	1.103	0.290–4.199	0.885	1.500	0.397–5.669	0.550
T stage	1.374	0.514–3.673	0.527	1.463	0.536–3.987	0.458
Status of lymph nodes	2.762	0.820–9.308	0.101	1.288	0.407–4.077	0.666
Smoking history	0.979	0.280–3.418	0.973	1.391	0.392–4.941	0.610
Alcohol history	1.251	0.376–4.155	0.715	0.875	0.250–3.060	0.835
PD-L1	2.737	1.201–6.240	0.017	1.914	0.849–4.317	0.118

## DISCUSSION

Usually, ESCC patients present with advanced disease or metastatic disease at diagnosis, who can not undergo esophagectomy. In addition, some patients can not undergo surgery because of the tumor location, severe cardiopulmonary dysfunction, or unwillingness of surgery. However, previous research on the association between PD-L1 in cancer cell and ESCC survival has been controversial and has focused on patients with esophagectomy. For patients without surgery, the prognostic value of PD-L1 remains to be determined. We analyzed prognostic significance of PD-L1 in patients without surgery for the first time. At present, immunotherapy has become an important treatment method for tumors with great potential. PD-L1 is the most commonly used predictor of immunotherapy. But in clinical applications, we have observed that not all PD-L1 positive patients can benefit from immunotherapy [21, 22]. Since only a few patients have good results after immunotherapy, screening beneficiary populations to guide treatment decisions has important clinical significance.

In the present study, we firstly analyzed all patients, then all patients were classified into two subgroups according to whether surgery was performed, one group of 37 ESCC patients receiving surgery and one group of 44 ESCC patients without surgery. In our study, PD-L1 proteins in cancer cells were positively expressed in 36 (44%) of 81 ESCC patients, 24 (65%) of 37 ESCC patients receiving esophagectomy, and 12 (27%) of 44 ESCC patients without surgery, respectively. The PD-L1 positive rate in surgical patients was obviously higher than those in non-surgical patients. In ESCC patients, multiple studies [23–25] have described that PD-L1 positive rate was between 18.9% and 79.7%. The differences between these researches may be due to discrepancy in the IHC evaluation methods and PD-L1 antibodies. On the contrary, Hirsch FR et al. [26] thought that although the PD-L1 assessment methods were different, the researchers emphasized that the results by Dako22C3 assay or Dako 28-8 assay were consistent, so this may have little effect on the results. In our study, we considered that the inconsistent expression rate may be due to different sources of specimens. For patients undergoing esophagectomy, we evaluated resection specimens, while for patients without surgery, we evaluated gastroscopic biopsy specimens.

Among all ESCC patients, there were no obvious associations between PD-L1 and alcohol history, smoking history, primary tumor location, age, sex, T stage, status of lymph nodes, M stage and TNM stage. Similarly, stratification analysis found that there were no obvious associations between PD-L1 and clinical and pathological features in both groups, respectively. These results were consistent with some studies [27, 28]. Wan-Ting Huang et al. [27] found that PD-L1 expression has no significant relationship with age, histological grade, primary tumor site, the clinical stages of T staging, N staging, and TNM staging. Sha Zhou et al. [28] showed that PDL1 positivity was not related with tumor location, T staging, alcohol history, age, sex, N staging and TNM staging. These observations indicated that PD-L1 expression was not affected by clinical and pathological characteristics.

For ESCC patients without surgery, associations between DCR with clinicopathological characteristics were also analyzed. In our study, we found that there were statistical associations between DCR with Tumor location (*P* = 0.046), TNM stage (*P* = 0.038) and PD-L1 expression (*P* = 0.032). DCR (78%) in the PD-L1 negative group was significantly better than those (42%) in the PD-L1 positive group. However, there were no apparent correlations between DCR and smoking history, alcohol history, age, sex, T stage and status of lymph nodes. Similarly, Wan-Ting Huang et al. [27] reported that pathologically complete responses (pCR) of PD-L1 negative patients after neoadjuvant chemoradiotherapy was higher than those of PD-L1 positive patients. Likely, Sha Zhou et al. [28] reported that patients with high PD-L1 expression had an obviously lower pCR rate. This was helpful to distinguish between responders and non-responders, thereby providing more appropriate treatment options.

In 81 ESCC patients, we did not find a significant association between PD-L1 and 3-year OS and 3-year PFS. Consistent with previous reports [18, 19], the expression of PD-L1 was not significantly correlated with patient's prognosis in ESCC patients underwent neoadjuvant treatment and esophagectomy. Differently, Qiao Wang et al. [15] reported that positive PD-L1 was statistically correlated with worse OS (*P* = 0.010) in ESCC patients received radical esophagectomy. And also, Jing-Jing Zhao et al. [16] reported that PD-L1 negative patients have longer OS than PD-L1 positive patients (*P* = 0.005) in ESCC underwent surgical resection. Furthermore, among the 378 patients with advanced ESCC who did not receive neoadjuvant chemoradiation and directly underwent radical esophagectomy, PD-L1 positive patients had worse disease-free survival (HR = 1.436, *P* = 0.009) [17]. Interestingly, in patients with ESCC undergoing postoperative adjuvant radiotherapy, Chenxue Jiang et al. [14] indicated that high expression of PD-L1 was correlated with a favorable prognosis. Similarly, Matteo Fassan et al. [29] discovered that PD-L1 positive patients had significantly higher pCR in patients with neoadjuvant chemoradiotherapy and surgery (*P* = 0.004). These results may show the prognostic role of PD-L1 in tumor cells was variable in different treatment methods. Thus stratification analysis was performed according to whether surgery was performed. The Kaplan-Meier method and log-rank test indicated that PD-L1 positive expression was significantly correlated with inferior 3-Year OS and PFS in 44 ESCC patients without surgery. In the Cox univariate and multivariate model, the PD-L1 positive expression was an independently poor prognosticators for inferior OS. For ESCC patients without surgery, the positive PD-L1 expression was correlated with inferior survival and increased risk of recurrence. However, in 37 ESCC patients receiving surgery, there was no obvious relationship between the positive expression of PD-L1 and 3-year OS and PFS. These findings indicated the impact of the immune predictors on the prognosis may vary with different treatment options, which was of great significance for the formulation of personalized treatment strategies, thereby provide a theoretical basis for provide a theoretical basis for combining conventional therapy with anti-PD-L1 immunotherapy currently recommended.

The present study has several limitations. First, our study was retrospective and the patient number was relatively small. Second, the expression of PD-L1 in immune infiltrating cells had not been assessed and we can not differentiate the central tumor from the invasion front because sample obtained by endoscopic biopsy was too small to have sufficient cells assessed. Besides, the discrepancy of positive PD-L1 expression rate between two groups may have resulted from different sources of specimens.

## CONCLUSIONS

In conclusion, our research revealed the prognostic role of PD-L1 expression in cancer cells may be variable with different treatment methods, which was of great significance to the development of personalized treatment plans. For ESCC patients without esophagectomy, the positive PD-L1 expression was correlated with inferior DCR and poor prognosis, and increased risk of recurrence. Consequently, PD-L1 may be used as an independent prognostic factor and provide a theoretical basis for combining conventional therapy with immunotherapy targeting PD-L1 to reach better therapeutic effect in ESCC patients without esophagectomy.

### Data availability statement

Some or all data during the study are available from the corresponding author by request. (Immunohistochemistry datas, OS and PFS).
